# Traumatic tricuspid valve regurgitation: A two case series

**DOI:** 10.1016/j.tcr.2021.100593

**Published:** 2021-12-23

**Authors:** A. Eranki, C. Villanueva, A. Wilson-Smith, P. Seah

**Affiliations:** Department of Cardiothoracic Surgery, John Hunter Hospital, Newcastle, Australia

**Keywords:** Trauma, Tricuspid valve injury, Blunt cardiac injury, Cardiac trauma, Cardiothoracic surgery

## Abstract

Traumatic tricuspid valve injury is rare, accounting for 0.02% of traumatic injuries. The majority of cases result from blunt force trauma to the chest, however penetrating injuries have been documented in literature. Patients' can be in the full spectrum of disease, from asymptomatic to cardiogenic shock. Indications for surgery include right heart failure or evidence of right heart volume overload in the setting of significant tricuspid regurgitation. Early surgical repair is warranted to preserve right ventricular function. Surgery also needs to be planned in conjunction with the patients' other injuries. In some cases, it may be beneficial for surgery to be delayed whilst the patient is closely observed, in order for the patient to recover from concomitant injuries. We report two cases of tricuspid regurgitation in the context of blunt trauma, and our approach to the management of these patients.

## Introduction

Traumatic tricuspid valve regurgitation (TTR) was first reported by Williams in 1829, with the surgical correction performed by Cooley in the late 1950's [1], [2]. It is rare, and accounts for only 0.02% of traumatic injuries, however this may be underdiagnosed [3], [4] In terms of mechanism, blunt trauma is responsible for the majority of cases [3]. Acute symptoms can be initially subtle, and patients may present years later with heart failure [5], [6]. Increased clinical awareness, use of biomarkers and widespread use of echocardiography has resulted in an increased diagnosis of TTR and earlier diagnosis [7]. Early surgical repair is warranted to preserve right ventricular function [8], [9], [10]. Indications for early surgery include severe to torrential regurgitation, associated with clinical and echocardiographic evidence of right ventricular strain. Repairing the valve is preferable, however replacement of the valve is feasible if repair is not an option. Delay in surgical management can lead to right ventricular failure, making repair less feasible [11], [12]. In the context of trauma, patients also present with multisystem injuries. The timing of surgery should take into account the patients' other injuries [12].

We describe two patients with traumatic tricuspid injury who were managed at the same institution by a single surgeon. We outline our approach to the management of TTR, especially in the context of other traumatic injuries. We also performed a narrative review of similar cases in literature, and offer an algorithm to the approach of TTR.

## Case 1

**Fig. 1:**
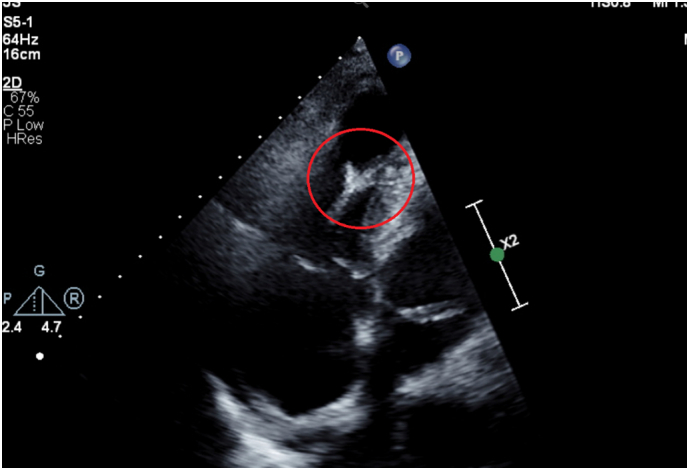
Apical four chamber view demonstrating partially avulsed papillary muscle.

A 62-year-old male was the driver of a stationary car hit from behind by a truck at high speed with significant cabin intrusion. On arrival, he was hemodynamically unstable. He had facial injuries, bilateral pulmonary contusions, bilateral rib fractures, pneumopericardium and a grade 5 splenic injury. Given the presence of significant pneumopericardium and hypotension, he underwent a transthoracic echocardiogram (TTE) which demonstrated moderately impaired right ventricular function with partial papillary muscle avulsion affecting the anterior and septal tricuspid leaflets. This resulted in moderate to severe tricuspid regurgitation (TR) (Fig. 1). There was no evidence of tamponade. Initial biochemistry demonstrated an elevated troponin level of 13,000 ng/L. He underwent an exploratory laparotomy, splenectomy and transferred to the intensive care in a stable condition. He remained intubated for a period of 12 days, complicated by a ventilator associated pneumonia.

Surgery for his tricuspid valve was initially considered on an urgent basis, however on serial examination there was no evidence of right heart failure or hemodynamic compromise. His right ventricular dysfunction was likely cardiac contusion. Given his concomitant chest injuries, recent splenectomy, poor ventilation and myocardial contusion, the decision was made to delay surgery, to mitigate his risks on cardiopulmonary bypass (CPB) and heparinisation. He was carefully monitored throughout his postoperative stay. A follow up TTE was performed 11 days after his original injury. There was no evidence of right ventricular failure with significant improvement in right ventricular function. Partial anterior papillary muscle rupture was again noted, with resultant moderate to severe TR.

**Fig. 2:**
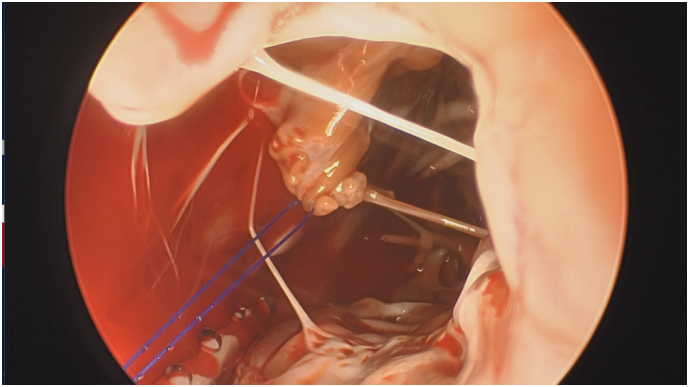
Partially avulsed papillary muscle suspended with GOR-TEX suture prior to repair.

**Fig. 3:**
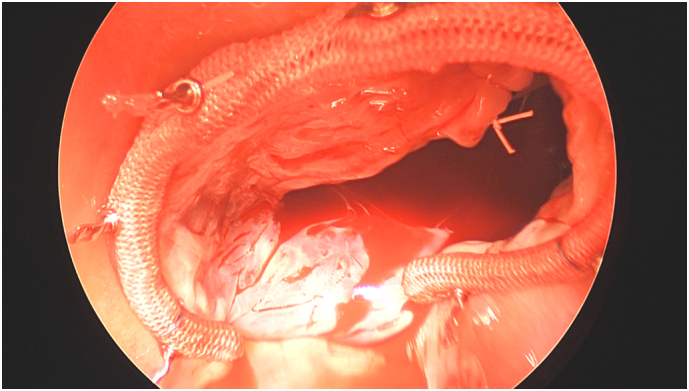
Annuloplasty ring and repair.

The patient was seen in clinic a month later after he completed a period of rehabilitation. A repeat TTE demonstrated normal right ventricular size with an ejection fraction 56%, but TR remained severe. Coronary angiography revealed mild disease. He underwent a tricuspid valve repair. On entry there were extensive adhesions of the pericardium predominantly over the right ventricle. After systemic heparinisation and placing the patient on CPB, right atriotomy revealed a partially ruptured papillary muscle causing prolapse of the anterior tricuspid valve leaflet. A 4-0 GOR-TEX suture was placed through the partially ruptured papillary muscle and neochordae fashioned and secured to the anterior leaflet (Fig. 2). A 34 mm Medtronic Contour Annuloplasty ring was seated (Fig. 3). On weaning from CPB, transesophageal echocardiography demonstrated a competent valve with no leak. He was discharged postoperative day 6.

## Case 2

A 51-year-old female, restrained driver was involved in a high-speed head on collision. Her chest impacted the steering wheel and airbags were deployed. She was GCS 15 and hemodynamically stable. She had evidence of significant chest and upper abdominal injuries on secondary survey. Once in the emergency department, she underwent a whole-body CT scan demonstrating a fractured manubrium and bilateral rib fractures. There were no intracranial or intraabdominal injuries. A TTE was performed that revealed a rupture of the anterior tricuspid papillary muscle with torrential tricuspid regurgitation. The right ventricle was mildly dilated and hyperdynamic. There were no regional wall motion abnormalities. There were no features of heart failure on examination. Despite multiple rib fractures, her pain was well controlled with multimodal analgesia. Given the degree of tricuspid regurgitation and early signs of right ventricular volume overload, the decision was made to proceed to urgent surgery.

The patient was placed on CPB and the heart arrested. Intraoperative findings revealed a torn anterior papillary muscle. A wedge resection of the lateral anterior leaflet containing the ruptured papillary muscle and its attendant chords was performed. The defect was closed with a continuous 4-0 Prolene suture. A 28 mm Edwards annuloplasty ring was secured to complete the repair. The repair was competent, and the patient was weaned off CPB without complications. She was discharged postoperative day 6 and was subsequently followed up with mild TR. She remains well 12 years down the line.

A literature search was done to outline other similar cases of traumatic tricuspid regurgitation and these are outlined in Supplementary Table 1 [11], [12], [13], [14], [15], [16], [17], [18], [19], [20], [21], [22], [23], [24], [25], [26]. Five case series were also summarised in Supplementary Table 2 [5], [6], [8], [9], [10].

## Discussion

Blunt thoracic trauma accounts for up to 25% of trauma deaths [3]. Cardiac injury resulting from blunt chest trauma encompasses a large range of presentations and is usually found in combination with other organ injuries which tend to obscure the cardiac injury [9]. Of valvular injuries, left sided valves (mitral and aortic valves) are more likely to be affected [27]. Tricuspid valve injury is rarer, however due to the advent of TTE and increased clinical suspicion, its incidence is rising [3], [7].

The mechanism of injury in the majority of cases is blunt force trauma [28]. Motor vehicle accidents are most common cause [6], [8], [9], [10]. Other common mechanisms include a fall from height and assault [8], [10]. The pathophysiology of injury involves direct compression, decompression or acceleration/deceleration forces to the thorax and indirect compression from the upper abdomen [29]. This results in increased intracardiac pressures. The right ventricle is vulnerable to injury as it is adjacent to the sternum. Its vulnerability is compounded by the increased hydrostatic pressure during the isovolumetric contraction phase, causing avulsion of the tricuspid leaflets [29]. The mechanism of injury in both our patients was blunt force trauma to the chest, in keeping with existing literature [11], [17], [18].

A diagnosis may be missed acutely because of coexisting multisystem involvement and the subtleness of physical signs [16], [18], [19], [20], [22], [24]. Due to the compliant nature of the right ventricle, tricuspid regurgitation may be tolerated well in the early phase and patients may present sometime later in heart failure with a retrospective history of blunt force trauma [22], [24]. During the index admission, patients exhibit the full spectrum of disease, from cardiogenic shock to mild shortness of breath on exertion. Atrial fibrillation and a new right bundle branch block may also indicate the presence of traumatic tricuspid injury [9]. Our first patient had evidence of cardiac injury with raised troponin levels and significant pneumopericardium. An urgent TTE was performed to investigate his hemodynamic instability whereby the diagnosis was made. Our second patient had evidence of significant chest trauma on secondary survey. A TTE was performed on the basis of her significant chest injuries, revealing the injury to the tricuspid valve. ECG is the single best predictor of cardiac injury, whereby it is abnormal in 40 to 83% of patients [30]. Some studies have also shown troponin to be a useful screening tool however case reports demonstrate tricuspid injury in the presence of normal troponin levels [30]. Routine TTE is low yield in blunt force trauma in the setting of normal troponin level and ECG. In this instance, cardiac injury is almost always ruled out [30].

Clinically overt heart failure has been the traditional indication for surgery [14]. Early surgery is recommended to avoid irreversible right ventricular dysfunction and dilatation. Van son et al. noted that the only patient within their case series that did not develop right ventricular dysfunction was operated within a month of injury [9]. Furthermore, it is postulated that if surgery is delayed, the papillary muscles, chordae tendineae and involved leaflets are found to be in an atrophied state precluding valve repair [8]. Ma et al. noted this in a case series of 13 patients [8]. In case one, there was no evidence of right heart failure. Due to the patient's concomitant injuries, we opted to delay surgery to facilitate recovery. Our second patient had significant TR with early signs of right ventricular strain. We opted to proceed to surgery on an urgent basis. With this in mind, we offer an approach to the management of TTR, summarised in Supplementary Fig. 1.

Repair should be attempted as the cohort of patients is often young. Common lesions involving the tricuspid valve include chordal rupture, papillary muscle rupture and leaflet rupture. Maisano et al. reviewed 74 reported cases and found that chordal rupture (n = 41, 55.4%) was the most common cause of TR (with 31 anterior chordal rupture cases accounting for 75.6% of chordal ruptures) [31]. A variety of techniques have been reported for TTR [24]. The flail leaflet can be repaired by plication with or without resection, chordae can be replaced with neochordae and papillary muscles can be reconstructed [8], [24]. Other techniques have been outlined in literature. Alfiere et al. repaired five tricuspid valves by stitching together the middle point of the free edges of the tricuspid leaflets, producing a clover-shaped valve [5]. This demonstrated good medium-term outcome [5]. Other case series demonstrate a high rate of repair [8], [9]. Patients fare well, with only one case reporting failure of repair and subsequent repeat surgery with replacement. Our first patient underwent a papillary muscle repair and neochordae. Our second patient recovered well from a wedge resection of affected leaflet and chords. We opted to utilize an annuloplasty ring in both our patients.

## Conclusion

This case series highlights an uncommon cardiac injury due to blunt chest trauma. Early surgery is advantageous to minimize the development of right ventricular failure in the setting of severe or torrential regurgitation. The timing of surgery must take into account with the patients' other injuries. We recommend serial examination and close follow up with TTE in this instance.

The following are the supplementary data related to this article.

Supplementary Fig. 1 Clinical practice recommendation for traumatic tricuspid regurgitation. Supplementary Fig. 1

Supplementary Table 1/2. Image 1

## Declaration of competing interest

There are no conflicts of interest to disclose.
