# Supplementary material for: Traumatic tricuspid valve regurgitation: A two case series
Source: Trauma Case Rep. 2021 Dec 23;37:100593. doi: 10.1016/j.tcr.2021.100593 (PMC8760512; doi:10.1016/j.tcr.2021.100593)
Supplement: Supplementary Fig. 1 — Clinical practice recommendation for traumatic tricuspid regurgitation. [file mmc1.docx]

Traumatic injury to tricuspid valve

Severity of regurgitation

Torrential

Moderate - Severe

Mild

Any catastrophic injuries/contraindications to early surgery

Evidence of RV volume overload or right heart failure?

Repeat TTE in 4 – 6 weeks’ time and reassess

Yes

No

Yes

No

Early Surgery

Early Surgery

Serial clinical examination and repeat TTE

Consider surgery once recovered from injuries

**Supplementary Figure 1:** Clinical practice recommendation for traumatic tricuspid regurgitation.
