# Supplementary material for: Traumatic tricuspid valve regurgitation: A two case series
Source: Trauma Case Rep. 2021 Dec 23;37:100593. doi: 10.1016/j.tcr.2021.100593 (PMC8760512; doi:10.1016/j.tcr.2021.100593)
Supplement: Supplementary file 2 — Supplementary Table 1/2. [file mmc2.docx]

**Supplementary Table 1: Traumatic Tricuspid Repair/Replacement Case Reports.**

| Author | Title | Patient | Mechanism | Other Injuries | Diagnosis | Timing of surgery | TV injury | Technique | Outcome |
| --- | --- | --- | --- | --- | --- | --- | --- | --- | --- |
| [Mehrotra](https://www.ncbi.nlm.nih.gov/pubmed/?term=Mehrotra%20D%5BAuthor%5D&cauthor=true&cauthor_uid=23109764) et al ^13^ | Tricuspid Valve Avulsion after Blunt Chest Trauma | 68 Female | fell approximately 8 to 10 meters onto rocks | Not specified | Heart Failure: index admission | Urgent | Avulsed anterior and posterior leaflets prolapsing | TV replacement | Well |
| Dounis et al ^14^ | Traumatic tricuspid insufficiency: a case report with a review of the literature | 21 Male | MBA | Head injury - GCS 8, multiple rib fractures, hemopneumothorax, Lung contusion RHS | Heart failure: Index admission | Elective | Anterior leaflet flail | NR | Well |
| Meel et al ^11^ | A Case of Severe Tricuspid Regurgitation Related to Traumatic Papillary Muscle Rupture | 25 Male | MVA (Pt vs dashboard) | Bilateral hemopneumothorax | Murmur: Index admission | Urgent | Anterior leaflet flail | TV repair: papillary muscle repair, De Vega Annuloplasty | Well |
| Stoica et al ^15^ | Traumatic Tricuspid Valve Rupture after Blunt Chest Trauma - A Case Report and Review of the Literature | 38 Female | MVA | Bilateral open leg fractures, small haemorrhagic cortical contusion, left hemopneumothorax, right pneumothorax, liver contusion, | Haemodynamic instability in ICU: Index admission | Urgent | Anterior leaflet prolapse | TV replacement | Well |
| Gelves et al ^16^ | Severe Aortic and Tricuspid Valve Regurgitation after Blunt Chest Trauma: An Unusual Presentation | 43 Male | MBA | Traumatic brain injury, facial fractures, Aortic valve injury, cardiac contusion | Heart failure: representation one month later | Urgent | Anterior and posterior leaflet prolapse | AV repair and TV replacement | Well |
| Jin et al ^17^ | A Case of Traumatic Tricuspid Regurgitation Caused by Multiple Papillary Muscle Rupture | 19 Male | MBA chest vs handlebar | NR | Elevated Troponin: index admission | Urgent: | Anterior and septal leaflet prolapse | TV repair: papillary muscle repair and ring annuloplasty | Well |
| Tutun et al ^18^ | Post-traumatic tricuspid insufficiency: a case report | 18 Male | Bicycle accident: chest vs handlebar | Face, thoracic cage, diaphragm tear | represented 18 months later with palpitations, found on TTE | Urgent | Dilated annulus, anterior and posterior leaflet prolapse | TV replacement | Well |
| Konstantinidou et al ^19^ | Repair of Tricuspid Valve Leaflet With CardioCel Patch After Traumatic Tricuspid Regurgitation | 26 Male | MVA | Chest and abdominal injuries | Represented 1 year later with heart failure | Urgent | Anterior and posterior leaflet destruction, | TV repair: leaflet reconstruction and neochordae and annuloplasty | Well |
| Okada et al ^20^ | Improvement of the Left Ventricular Function after Tricuspid Valve Plasty for Traumatic Tricuspid Regurgitation | 61 female | MVA | Rib fractures, sternal fracture, R hemothorax | Represented with SOBOE 1 month after | Urgent | Anterior tricuspid valve prolapse | TV repair: neochordae and annuloplasty | Well |
| Caruso et al ^21^ | Traumatic tricuspid valve regurgitation: A challenging case report | 26 Male | Fall from height | Pneumothorax, Pericardial and pleural effusion | Drained pericardial effusion, found to have severe TR on TTE | Urgent | Anterior leaflet prolapse | TV repair: neochordae and annuloplasty | Failure of repair: anterior leaflet rupture - TV replacement |
| Emmert et al ^22^ | Severe traumatic tricuspid insufficiency detected 10 years after blunt chest trauma | 29 Male | MVA | Clavicle fracture, rib fractures | Systolic murmur on routine medical exam 10 years later TOE R/O endocarditis. No hx of fevers | Elective | Flail anterior leaflet | TV repair: neochordae and annuloplasty | Well |
| Reddy et al ^23^ | Traumatic Tricuspid Papillary Muscle and Chordae Rupture: Emerging Role of Three-Dimensional Echocardiography | 32 Male | MBA | Anterior chest wall injury, splenic laceration, pulmonary contusion | Systolic murmur : index admission | Urgent | Flail anterior leaflet, ruptured papillary muscle | TV repair | Well |
| Hirao et al ^24^ | Surgical repair of tricuspid regurgitation due to annular detachment caused by chest trauma | 45 Female | MVA | Cerebral contusion, pulmonary contusion, R NOF, | Represented 16 years later with SOBOE | Elective | Anterior leaflet prolapse and perforation of posterior leaflet, R atrial wall perforation | TV repair: neochordae and annuloplasty, suture repair of perforation | Well |
| Conaglen et al ^25^ | Acute Repair of Traumatic Tricuspid Valve Regurgitation Aided by Three-Dimensional Echocardiography | 54 Male | MVA vs Tree | Bilateral lung contusions, left rib fractures 1-6, R rib fractures 2 - 6, R clavicle fracture, L maxillary sinus fracture | Elevated Troponin: index admission | Urgent | Anterior papillary muscle rupture | TV repair with neochordae and annuloplasty | Well |
| Chu et al ^26^ | Repair of Traumatic Tricuspid Regurgitation | 30 Male | MBA | Periaortic haematoma, haemopericardium, liver laceration, | Found during emergent sternotomy (damage control surgery), elected not to repair, represented with heart failure | Elective | Perforated anterior leaflet, dilated annulus | TV repair with neochordae and annuloplasty | Well |
| Longfello et al^12^ | Traumatic Injury of the Tricuspid Valve—Navigating the Challenges in Diagnosis and Management | 38 Male | MVA | Open femur fracture, Open R patella fracture, Manubrial fracture, multiple rib fractures, haemothorax. | Worsening heart failure: index admission | Elective Once Recovered | Flail anterior leaflet | TV replacement | Well |

**Supplementary Table 2: Traumatic Tricuspid Repair/Replacement Case Series.**

| Authors | Title | # patients | Age range | Mechanism | Mechanism of TR | Repair/Replacement | Conclusion |
| --- | --- | --- | --- | --- | --- | --- | --- |
| Ma et al ^8^ | Surgical Treatment of Traumatic Tricuspid Insufficiency: Experience in 13 Cases | 13 | 17 - 53 (mean 40) | MVA/MBA 9 patients, fall 2 patients, explosion 1 patient, assault (blunt force) 1 patient | Anterior chordal rupture - 9, Anterior papillary muscle rupture - 4, Posterior chordal rupture - 2, Anular dilatation - 7, Anterior leaflet defect - 1 | 13/0 | Tricuspid valve repair techniques can often be performed for traumatic tricuspid insufficiency with low risk, allowing symptomatic relief with excellent midterm outcomes. Early operation should be emphasized to achieve good functional results and preserve the right ventricular function |
| Van Son et al ^9^ | Traumatic tricuspid valve insufficiency | 13 | 17 - 64 (median 39 years) | MVA 12, Explosion 1 | Anterior chordal rupture - 8, Anterior papillary muscle rupture - 4, Anterior leaflet tear -3, Septal leaflet injury - 1 | 5/8 | Good functional results can still be achieved many years after the onset of traumatic tricuspid valve insufficiency, earlier diagnosis and surgical treatment should increase the feasibility of tricuspid valve repair, prevent progressive deterioration of right ventricular function, and increase the possibility of maintaining late sinus rhythm in a greater number of patients |
| Alfieri et al^5^ | The “clover technique” as a novel approach for correction of post-traumatic tricuspid regurgitation | 5 | 27 - 75 years | MVA - 5 | Anterior leaflet prolapse - 5, posterior leaflet prolapse 3, annular dilatation 5 | 5/0 | the clover technique increased the feasibility of tricuspid valve repair in case of severe traumatic tricuspid valve insufficiency, leading to very satisfactory mid-term results even in the presence of complex lesions or dilatation and deterioration of the right ventricle |
| Zhang et al ^10^ | Surgical management of traumatic tricuspid insufficiency | 10 | 22 -63 (mean 49) | MVA 6, blows to chest 3, fall 1 | Anterior chordal rupture 8, Anterior papillary muscle rupture - 2, Anterior leaflet tear 1 | 5/5 | Early surgery is recommended for achieving a successful valve repair and preserving right ventricular function |
| Schuster et al ^6^ | Heterogeneity of traumatic injury of the tricuspid valve: A report of four cases | 4 | 20 - 63 | MVA - 4 | Anterior papillary muscle rupture - 2, Anterior chordal rupture 1, Septaal papillary muscle -1 | 4/0 | Because surgical treatment is required in most patients and since the diagnosis is often delayed, we believe that early echocardiographic evaluation is required in all patients with blunt chest trauma, in particular if clinical symptoms of right heart failure are present. |
